# Effects of physical activity on regulatory emotional self-efficacy, resilience, and emotional intelligence of nurses during the COVID-19 pandemic

**DOI:** 10.3389/fpsyg.2022.1059786

**Published:** 2022-12-09

**Authors:** Ruoshan Wu, Longjun Jing, Yang Liu, Huilin Wang, Jingyu Yang

**Affiliations:** ^1^School of Physical Education, Hunan University of Science and Technology, Xiangtan, China; ^2^China Athletics College, Beijing Sport University, Beijing, China; ^3^School of Business, Hunan University of Science and Technology, Xiangtan, China; ^4^Faculty of Economics, Chulalongkorn University, Bangkok, Thailand; ^5^Department of Medical Bioinformatics, University of Göttingen, Göttingen, Germany

**Keywords:** physical activity, regulatory emotional self-efficacy, emotional intelligence, resilience, nurse

## Abstract

The normalization of epidemic prevention and control has exacerbated nurses’ physical and mental stresses. The important role of physical activity in relieving nurses’ physical and mental stresses has received extensive attention from researchers in recent years. The purpose of this study was to investigate the influence of physical activity on the regulatory emotional self-efficacy, resilience, and emotional intelligence of nurses and explain their interactions. The present study adopted the cluster sampling method. From April to May 2022, a total of 500 nurses in six municipal hospitals in Changsha City were selected. Finally, 402 valid data samples were obtained. Afterward, AMOS 23.0 (by maximum likelihood estimation) was used to process the collected data and analyze the proposed hypotheses by using 5,000 bootstrap samples to test the mediating effects of the structural equation model. The results demonstrated that there are positive correlations between physical activity and resilience (standardized coefficients = 0.232, *p* < 0.001), resilience and regulatory emotional self-efficacy (standardized coefficients = 0.449, *p* < 0.001), and emotional intelligence and regulatory emotional self-efficacy (standardized coefficients = 0.330, *p* < 0.001). The positive influence of physical activity on emotional regulation self-efficacy is completely mediated by emotional intelligence and resilience (standardized indirect effect = 0.237, *p* < 0.01), and this explanatory power is far higher than any previous study (*R*^2^ = 0.49). The positive emotions generated by an individual’s physical activity have an important explanatory role for individuals who want to establish more emotional regulation self-efficacy, emotional intelligence, and psychological resilience.

## Introduction

Although the COVID-19 pandemic is greatly weakening, the nurses who are on the front lines of epidemic prevention have not relaxed. On the contrary, with the normalization of epidemic prevention and control in China, nurses have become the main force in applying epidemic prevention and control (Keshkar et al., 2021). Every time a major holiday begins or ends, hundreds of millions of people return to their hometowns, schools, and jobs. Nurses are often overwhelmed by having to conduct nucleic acid testing on people from all walks of life and constantly having to repeat boring work processes. This pressure was not only reflected in the fact that they needed to complete more than 8 h of work in protective clothing when the outdoor temperature exceeded 30°C (73°F) in summer, it was also reflected in the normalization of epidemic prevention and control likely to be continuing for a long time in the future. This form of work will become their norm. Nurses often suffer from excessive psychological pressures that affect their mental health and cause problems, such as job burnout, decreased happiness, and suicidal tendencies (Hamre et al., 2020). It is therefore important to know how to relieve these excessive psychological pressures on the nurses through certain means in the post-epidemic era to improve their mental health.

In recent years, the relationship between physical activity and individual mental health has received extensive attention from researchers (Chekroud et al., 2018). Research shows that people who are physically active have higher levels of mental health, and they tend to be less susceptible to mental illnesses, such as anxiety, depression, emotional disorders, and insomnia (Stafseth et al., 2022). Physical activity allows people to obtain an emotional experience that liberates them from repressed mental states and then plays a role in eliminating negative emotions (Tamminen and Bennett, 2017). The extended theory of positive emotions believes that an individual’s long-term emotional experience will be transformed into an emotional ability (Fredrickson, 1998). When people are in a negative and repressed emotion for a long time, this actually indicates that they are losing the ability to experience positive emotions (Fredrickson, 1998). When in the midst of a positive emotional experience, a positive emotion actually transforms into an ability to experience positive emotions while also enhancing the ability to resist negative emotions (Fredrickson, 2001). Relevant studies have shown that individuals who participate in physical activity for a long time have higher emotional intelligence, and their perceptions and control of emotions will be significantly enhanced (Frazier and Nagy, 1989). Physical activity can make people gain a sense of positive emotional experiences so that an individual’s emotions can be released and relaxed during exercise, thereby reducing or eliminating the impact of negative emotions on the individual’s mental health (Wang et al., 2020).

For the nurses, their occupational characteristics cause their mental health to be affected by more uncertain factors than the general population, such as doctor–patient relationship, working environment, work and rest time, especially in the context of the normalization of epidemic prevention and control, The dull and single work process and severe work environment have led to the accumulation of nurses’ negative emotions (Jeung et al., 2018; Alsaqri et al., 2021). When the long-term accumulated psychological pressure cannot be effectively relieved, it will increase the risk of individuals suffering from mental illness (Lorente et al., 2021). Although the psychological pressure generated by the external environment exists objectively, the ability of individuals to resist negative emotions varies from person to person and is affected by many factors, such as emotional regulation, emotional intelligence, and psychological resiliency (Labrague and De los Santos, 2020). It is worth noting that most of the previous studies focused on the descriptive level of the relationship between physical activity and individual mental health, but few studies have paid attention to how physical activity affects individuals’ mental health.

Emotional regulation of self-efficacy, emotional intelligence, and resilience has been regarded as the major predictor of individual mental health in recent years (Young et al., 2011). An individual’s ability to regulate emotions was once considered to be an important channel for stress relief (McEwen et al., 2015). Emotion regulation, resilience, and social support play a combined and important role in the maintenance of the mental health of frontline health-care workers during the COVID-19 pandemic (Fino et al., 2021). Regulatory emotional self-efficacy, as an important predictor of individual emotional regulation ability, has received extensive attention from researchers in recent years. Studies have shown that regulatory emotional self-efficacy is significantly related to prosocial behavior, mental health, self-esteem, subjective well-being, and other factors (Bandura et al., 2003). Other studies have shown that emotional regulation self-efficacy, emotional intelligence, and resilience can significantly affect individual mental health, and physical activity has an important role in promoting individuals’ mental health, but little attention has been paid to how physical activity affects emotional regulation self-efficacy, emotional intelligence, and resilience, and how they interact to promote an individual’s mental health (Paluska and Schwenk, 2000).

This study attempts to achieve the following objectives: (1) to investigate the effects of nurses’ physical activity on their emotional intelligence, resilience, and regulatory emotional self-efficacy; (2) to explore the impact mechanism of physical activity on emotional intelligence, resilience, and regulatory emotional self-efficacy; (3) to explore whether and how emotional intelligence, resilience, and regulatory emotional self-efficacy interact with each other; and (4) to propose suggestions for improving the physical activity of nurses.

The contributions of this study are as follows: First, by exploring the effect of physical activity on emotional intelligence, psychological resilience, and regulatory emotional self-efficacy, the research on the influence mechanism of physical exercise on individual mental health was expanded. Second, this study adopted the extended broaden-and-build theory of positive emotions, which highlighted the important explanatory role of positive emotions generated by individuals participating in physical activities to build more regulatory emotional self-efficacy, intelligence, and resilience. Third, this study expanded the research on the micro-influence mechanism of emotional regulation self-efficacy. As in previous studies, regulatory emotional self-efficacy is usually used as a mediator and moderator variable affecting the results of the study, but in this study, regulatory emotional self-efficacy will be the target variable.

## Literature interviews and hypotheses development

### Broaden-and-build theory of positive emotions

Fredrickson (1998). proposed the broaden-and-build theory of positive emotions. Through the research on the four positive emotions of happiness, interest, satisfaction, and love Fredrickson (2001) believed that positive emotions could broaden the thought-action ability of individuals. Fredrickson pointed out that positive emotions can effectively expand the individual’s attention span, creative thinking, and cognitive ability.

In subsequent research, Tugade and Fredrickson (2004).proved that positive emotions play a positive role in relieving negative emotions, improving resilience, and promoting emotional health. Relevant studies showed that physical activity plays an important role in promoting an individual’s physical and mental health and creating positive emotions (Bouchard et al., 1994; Gehlhar et al., 2022). Based on the extended broaden-and-build theory of positive emotions, this study attempts to discuss how physical activity affects emotional intelligence, resilience, and regulatory emotional self-efficacy.

### Physical activity and resilience

In recent years, there has been a prevailing view in the mass media and academia that the psychological benefits of physical activity may equal or even exceed the physical benefits (Smith et al., 1996). Specifically, first, people who regularly participate in sports have higher levels of mental health and are less susceptible to negative emotions such as depression, anxiety, and stress (Scully et al., 1998). Second, exercise can enhance an individual’s emotional state. People who regularly participate in sports have a more optimistic attitude, a higher cognitive level, and a more positive emotional experience (Frazier and Nagy, 1989). Third, exercise can effectively shape personality, and studies have shown that physical activity is positively correlated with self-esteem, emotional intelligence, and self-efficacy (Wang et al., 2020; Aouani et al., 2022; Liu and Qiang, 2022).

Resilience, as a personality trait, reflects an individual’s ability to cope successfully or adapt well in the face of adversity (Masten, 2001). Individuals with high resilience have a more optimistic attitude and more positive emotions. They are often able to face problems head-on and are good at using positive emotions, so they also have a higher sense of well-being, a higher level of mental health, and a stronger ability to deal with emergencies (Chan et al., 2013). In the formation and development of resilience, the consensus view is that resilience is affected by protective factors and risk factors (Kumpfer, 2002). Protective factors include individual factors, such as self-efficacy, intellectual function, attribution style, and self-esteem, as well as non-individual factors such as family environment and social support (Masten and Coatsworth, 1998). When an individual encounters adversity, the body responds quickly to the current environment through protective factors, so that the body can adapt well to the current environment or quickly recover from the traumatic state. Risk factors refer to certain biological, psychological, cognitive, or external environmental factors that hinder the normal development of an individual, such as poverty, physical illness, and the crisis of abuse and violence.

To sum up, there are the following connections between physical activity and resilience: First, both physical activity and resilience are important promoting factors for individuals’ mental health. Second, physical activity appears to promote protective factors of resilience such as self-esteem and self-efficacy, while physical activity enhances social support by building wider social networks. Third, individuals enhance their physical, psychological, cognitive, and other abilities by participating in sports, thus reducing the impact of risk factors on psychological resilience. Based on this, this study proposes the following hypotheses:

*Hypothesis* 1 *(H1)*: Physical activity has a positive impact on resilience.

### Emotional intelligence, resilience, and regulatory emotional self-efficacy

According to the emotional intelligence competency model of Mayer et al. (1999)., emotional intelligence is mainly composed of accurately perceiving emotions in oneself and others, using emotions to facilitate thinking, understanding emotional meanings, and managing emotions. Related research suggests that people with high emotional intelligence may have better levels of mental health and well-being because they have a better ability to regulate stress and flexibility to improve the effects of negative emotions (Ciarrochi et al., 2002). People with high emotional intelligence have a better ability to understand, control, and manage emotions, and they seem to be more successful at avoiding interpersonal squabbles and fights, and thus have better social support and relationships (Ciarrochi et al., 2002). People with high emotional intelligence have a better ability to perceive and use emotions, as they are good at regulating their emotions, and thus they have a more positive emotional state (Salovey et al., 2009; Sharma et al., 2016).

As a branch of self-efficacy, regulatory emotional self-efficacy refers to a degree of self-confidence of individuals in their emotion regulation ability, which affects the process of emotion regulation and is the basis of emotional competence (Bandura et al., 2003). In other words, regulatory emotional self-efficacy is the expectation of behavioral subjects on their emotional regulation ability, which is based on self-evaluation and represents a cognitive state of one’s ability. Research by Kavanagh and Bower (1985). showed that positive emotions are a significant predictor of self-efficacy. Research by Caprara et al. (2003). pointed out that individuals with higher self-efficacy in regulating negative emotions have higher levels of emotional stability.

Taken together, emotional intelligence, resilience, and regulatory emotional self-efficacy appear to be highly correlated in theory. On the one hand, emotional intelligence is the individual’s ability to control emotions. To a certain extent, emotional intelligence provides a cognitive judgment for regulatory emotional self-efficacy and is the basis for regulatory emotional self-efficacy. While regulatory emotional self-efficacy seems to be predicted by positive emotions, higher emotional intelligence also seems to predict higher positive emotions. Emotional intelligence seems to be a predictor of regulatory emotional self-efficacy. On the other hand, according to the related definition of resilience, self-efficacy is one of the protective factors in resilience, and higher resilience predicts higher positive emotions (Kumpfer, 2002). Therefore, regulatory emotional self-efficacy, which is also affected by positive emotions, seems to be related to psychological resilience. In addition, a significant positive correlation between emotional intelligence and resilience has been demonstrated (Akbari and Khormaiee, 2015; Jayalakshmi and Magdalin, 2015). Based on this, this study proposes the following hypotheses:

*Hypothesis* 2 *(H2)*: Resilience has a positive impact on regulatory emotional self-efficacy.

*Hypothesis* 3 *(H3)*: Emotional intelligence has a positive impact on regulatory emotional self-efficacy.

### The mediating roles of emotional intelligence and resilience

According to the broaden-and-build theory of positive emotions, positive emotions promote the establishment of various personal resources (e.g., material, intellectual, and social resources) by broadening the individual’s ability to think and act, and then have an impact on the formation and development of an individual’s personality (Fredrickson, 1998). It should be pointed out that Fredrickson’s previous research only clarified that positive emotions can have an impact on individual elements of an individual’s personality such as creative thinking, cognitive ability, and resilience. But Fredrickson did not explain whether these factors interacted under the influence of positive emotions. Previous research has demonstrated that emotional intelligence, resilience, and regulatory emotional self-efficacy may be affected by positive emotions (Fredrickson, 2001). Based on this, it is the focus of this study to explore how they are affected by positive emotions and how they interact with each other.

Relevant studies have demonstrated that physical activity could promote positive emotions in individuals, as there is a significant relationship between physical activity and emotional intelligence and a significant relationship between emotional intelligence and resilience (Jayalakshmi and Magdalin, 2015; Wang et al., 2020). Thus, physical activity can have an impact on resilience through emotional intelligence. If there is a significant relationship between resilience and regulatory emotional self-efficacy, can emotional intelligence affect regulatory emotional self-efficacy? Assuming that physical activity, emotional intelligence, resilience, and regulatory emotional self-efficacy are all directly linked, can emotional intelligence and resilience mediate the relationship between physical activity and regulatory emotional self-efficacy? Based on this, this study proposes the following hypotheses:

*Hypothesis* 4 *(H4)*: Emotional intelligence mediates the relationship between physical activity and resilience.

*Hypothesis* 5 *(H5)*: Resilience mediates the relationship between emotional intelligence and regulatory emotional self-efficacy.

*Hypothesis* 6 *(H6)*: Emotional intelligence and resilience mediate the relationship between physical activity and regulatory emotional self-efficacy.

**Figure 1:**
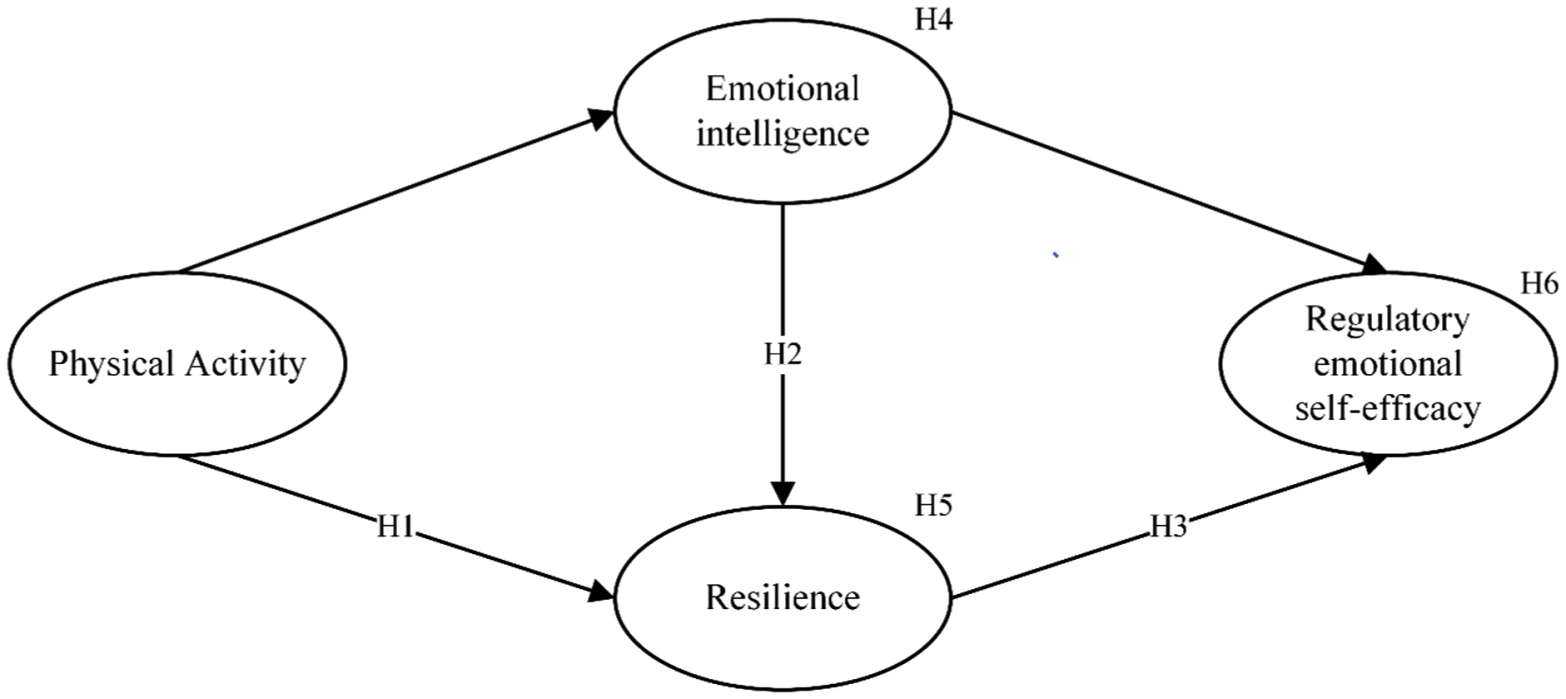
The hypothesized model.

A summary of all the hypotheses in this study is shown in Figure 1.

## Materials and methods

### Procedure

In this study, the cluster sampling method was adopted, and the samples were drawn from the groups as the sampling unit, and each group was required to have good representativeness. According to the geographical distribution of the six municipal districts of Changsha, the researchers randomly selected one municipal hospital in each municipal district and finally took the nurses at the six municipal hospitals as the research objects. From April to May 2022, the researchers received the help of the relevant management personnel of the hospital’s medical department, and through them, the nurses in each department were given the task of filling out the online questionnaire. In the end, a total of 500 questionnaires were distributed and 402 valid questionnaires were recovered for a recovery rate of 80.4%.

### Measures

This study measured physical activity by using three items on the scale of Andersen et al. (2010). and measured resilience by using six items on the scale of Campbell-Sills and Stein (2007). Emotional intelligence was measured using six items on the Law et al. (2004) scale, originally developed to measure the emotional intelligence of respondents in Eastern countries. Four items were selected from the study by Caprara et al. (2008) to measure regulatory emotional self-efficacy. These four scales were all measured using a five-point Likert scale, where responses ranged from 1 (i.e., strongly disagree) to 5 (i.e., strongly agree). All items are shown in Table 1.

**Table 1:** Reliability and validity tests.

Items	Loadings	Cα	AVE	CR
*Physical activity (PA)*		0.792	0.561	0.792
PA1: In your leisure time, how often do you spend per week doing light physical activity such as walking, light cleaning, and yoga?	0.824			
PA2: In your leisure time, how often per week do you engage in gardening, carrying loads upstairs, or doing moderately strenuous sports week doing light physical activity?	0.729			
PA3: In your leisure time, how often per week do you engage in strenuous sports and conditioning exercises such as running, jogging, soccer, tennis, or similar activities?	0.687			
*Resilience (RE)*		0.893	0.584	0.893
RE1: I am not easily discouraged by failure.	0.766			
RE2: I can stay focused under pressure.	0.752			
RE3: I can achieve goals despite obstacles.	0.812			
RE4: I tend to bounce back after illness or hardship.	0.754			
RE5: Coping with stress can strengthen me.	0.727			
RE6: I can deal with whatever comes.	0.771			
*Emotional intelligence (EI)*		0.792	0.574	0.800
EI1: I have good control of my own emotions.	0.798			
EI2: I can control my temper so that I can handle difficulties rationally.	0.823			
EI3: I am a self-motivating person.	0.639			
*Regulatory emotional self-efficacy (RES)*		0.825	0.555	0.830
RES1: I can try to avoid negative experiences because I fail to achieve my goals.	0.806			
RES2: I can reduce the upset when I do not get the appreciation, I feel I deserve.	0.786			
RES3: I can avoid getting upset when others keep giving me a hard time.	0.809			
RES4: I feel gratified about overachieving what I set out to do.	0.544			

All standardized loadings are significant at the 0.001 level.

To adapt to the specific research field and Chinese cultural background, the researchers made certain adjustments to the items of the scales. A pilot test was used to ensure the reliability of the adjusted scale (Kimberlin and Winterstein, 2008). Taking the nurses of a municipal hospital as the survey object, the researchers distributed 60 questionnaires using the convenience sampling method and recovered 54 valid questionnaires. The results showed that Cronbach’s alpha coefficients were all greater than 0.8, indicating that the measuring instruments had good internal consistency (Fornell and Larcker, 1981).

### Reliability and validity

Reliability is measured by Cronbach’s α coefficient and composite reliability (CR) coefficient, recommended by Fornell and Larcker (1981). As shown in Table 2, the lowest value of Cronbach’s α coefficient in all variables is 0.792, which is above the minimum value of 0.7 recommended by Hair et al. (2012). Therefore, the reliability of all variables is ideal. The convergent validity was evaluated by two indicators’ factor loading and average variance extracted (AVE; Fornell and Larcker, 1981). Table 2 shows that the lowest values of factor loading and AVE in all measurement items are 0.544 and 0.555, respectively, both of which are higher than the recommended value of 0.5 suggested by Fornell and Larcker (1981). Therefore, all variables have high convergent validity. Finally, the discriminant validity is verified by comparing the square root of AVE and the correlation coefficient of each variable. The results from Table 3 show that the square root of the AVE of each construct is greater than the correlation coefficients, which meets the requirement for the existence of discriminant validity suggested by Fornell and Larcker (1981).

**Table 2:** Discriminant validity test.

Construct	PA	RE	EI	RES
PA	**(0.749)**			
RE	0.386^**^	**(0.764)**		
EI	0.262^**^	0.676^**^	**(0.758)**	
RES	0.180^**^	0.570^**^	0.524^**^	**(0.745)**

The square root of the average variance extracted (AVE) is in diagonals (bold); off diagonals are Pearson’s correlations of constructs. ^**^p < 0.01.

**Table 3:** Participant profile (*N* = 402).

Profiles	Survey	2020 Statistical bulletin of China’s health development^a^
*Respondent age (%)*		
18–25	10.6	
26–35	52.9	
36–45	24.2	
≥ 45	12.3	
*Respondent gender (%)*		
Male	4.3	3.6
Female	95.7	96.4
*Respondent education level (%)*		
Below high school	2.1	1.0
High school/Vocational school	10.8	13.8
College/University	79.3	85.2
Master or Ph.D.	7.8	

a National Health Commission (2022).

### Data analysis

This study used structural equation modeling (SEM) with AMOS 23.0 to analyze the proposed model. SEM is often used to evaluate latent variables on measurement models and to test hypotheses between latent variables on structural models (Hair et al., 2012). This study adopted the two-step modeling approach proposed by Anderson and Gerbing (1988). First, the researchers tested the reliability and effectiveness of the instrument as shown in “Reliability and validity”, and the average value of the Cronbach α coefficient in all variables was 0.792, indicating good reliability and effectiveness of the instrument. Second, the researchers used the maximum likelihood estimation method to verify the significant relationship between physical activity, regulatory emotional self-efficacy, resilience, and emotional intelligence as independent variables. Third, the researchers used 5,000 bootstrap samples to test the indirect effects between physical activity and regulatory emotional self-efficacy. Finally, evaluated the validity of the model, and measure the fit coefficients and path coefficients of the hypothetical model.

## Results

### Participants

Table 1 lists the demographic characteristics of the 402 respondents: (1) In terms of age, 63.5% were 18–35 years old, and the entire sample tends to be younger in age; (2) In terms of gender, the proportion of males was 4.3%, female accounted for 95.7%; (3) In terms of education level, the majority of respondents were college graduates (79.3%). The survey results were close to the 2020 Statistical Bulletin of China’s Health Development (National Health Commission, 2022).

### Structural path model

**Figure 2:**
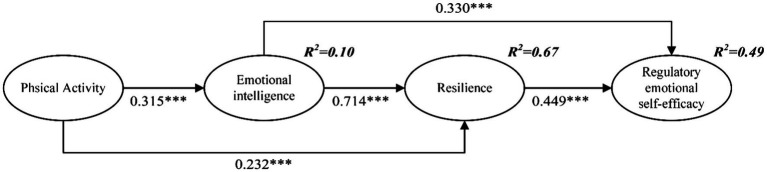
Structural path model. ^***^p < 0.001. Standardized coefficients are reported.

Referring to the test parameters commonly used in previous studies (Jackson et al., 2009). and the suggested values of Hair et al. (2012). the results show that the data (*χ*^2^/*df* = 2.433, GFI = 0.934, NFI = 0.928, CFI = 0.956, TLI = 0.946, IFI = 0.956, RMSEA = 0.060) has a good fit with the structural model. The results of the structural path model are shown in Figure 2. There are positive correlations between physical activity and resilience (standardized coefficients = 0.232, *p* < 0.001), resilience and regulatory emotional self-efficacy (standardized coefficients = 0.449, *p* < 0.001), and emotional intelligence and regulatory emotional self-efficacy (standardized coefficients = 0.330, *p* < 0.001) and all are statistically significant, so H1, H2, and H3 were supported. The relationship of physical activity to resilience, emotional intelligence, and regulatory emotional self-efficacy appeared to be mediated by emotional intelligence and resilience. Although the effect of physical activity on regulatory emotional self-efficacy was not statistically significant, the effect of physical activity on regulatory emotional self-efficacy appeared to be mediated by emotional intelligence and resilience.

### Mediation test

For testing the mediation effect, this study followed the suggestion of Bollen and Stine (1990)., and the bootstrapping method was used. The 95% confidence interval results for the 5,000 bootstrap samples are shown in Table 4. All *Z* values are greater than 1.96, and there is no zero value in the 95% confidence interval. There was a significant mediating effect between physical activity and resilience through emotional intelligence (standardized indirect effect = 0.165, *p* < 0.001), and H4 was supported. There was a significant mediating effect between emotional intelligence and regulatory emotional self-efficacy through resilience (standardized indirect effect = 0.338, *p* < 0.001), and H5 was supported. There was a significant mediating effect between physical activity and regulatory emotional self-efficacy through resilience and emotional intelligence (standardized indirect effect = 0.237, *p* < 0.01), and H6 was supported.

**Table 4:** Standardized direct, indirect, and total effects.

	Point Estimate	Product of coefficients		Bootstrapping				
				Percentile 95% CI		Bias-corrected 95% CI		Two-tailed significance
		SE	*Z*	Lower	Upper	Lower	Upper	
*Direct effects*								
PA → RE	0.171	0.038	4.500	0.095	0.245	0.102	0.249	0.000(^***^)
EI → RES	0.348	0.112	3.107	0.142	0.584	0.135	0.577	0.002(^**^)
*Indirect effects*								
PA → RE	0.165	0.039	4.231	0.093	0.245	0.095	0.251	0.000(^***^)
PA → RES	0.237	0.046	5.152	0.152	0.335	0.157	0.341	0.000(^***^)
EI → RES	0.338	0.092	3.674	0.162	0.518	0.172	0.531	0.002(^**^)
*Total effects*								
PA → RE	0.336	0.048	7.000	0.244	0.430	0.248	0.435	0.000(^***^)
PA → RES	0.229	0.049	4.673	0.060	0.253	0.064	0.256	0.002(^***^)
EI → RES	0.686	0.065	10.554	0.566	0.821	0.568	0.823	0.000(^***^)

Standardized estimation of 5,000 bootstrap samples; ^**^p < 0.01, ^***^p < 0.001.

## Discussion

### Contributions

This study makes the following contributions to the study of regulatory emotional self-efficacy. It explores the effect of physical activity on the regulatory emotional self-efficacy of nurses. At the same time, emotional intelligence and psychological resilience were added as mediators providing a clearer path for the impact of physical activity on regulatory emotional self-efficacy. The findings showed that the positive effect of physical activity on regulatory emotional self-efficacy was mediated by emotional intelligence and resilience. This explains 49% of the variance in regulatory emotional self-efficacy, which was much higher than the 27% explanatory power of previous studies (Chan, 2004). According to previous research, scholars defined regulatory emotional self-efficacy as a cognitive judgment of one’s emotion regulation ability. From this perspective, both the individual’s internal cognitive ability and the influence of the external environment will affect the individual’s cognitive state. Emotional intelligence, as the ability to perceive, use, understand and manage emotions, essentially exerts an influence on self-efficacy in emotion regulation by changing the individual’s intrinsic cognitive ability to emotions. Mayer (2015). sees emotional intelligence as a mental ability, which is exactly in line with this point of view; while resilience affects regulatory emotional self-efficacy through an experiential environment from the outside world.

Previous studies on resilience found that individual resilience needs to be improved in the face of constant adversity, which is manifested as an empirical adaptation process. In other words, when different individuals face the same situation, individuals with similar experiences tend to have higher regulatory emotional self-efficacy. This study attempts to use an extended broaden-and-build theory of positive emotions to explain the effects of physical activity on emotional intelligence, resilience, and regulatory emotional self-efficacy. Positive emotions can motivate individuals to act to promote individuals to build material, intellectual, social, and other resources, among which intellectual resources are the focus of this research (Fredrickson, 1998). Positive emotions can promote the establishment of individual intellectual resources. From this point of view, emotional intelligence, resilience, and regulatory emotional self-efficacy, as important individual intellectual resources, may also be affected by positive emotions. Fredrickson’s (2001) subsequent research confirmed this view by studying the impact of positive emotions on individual intellectual factors such as creative thinking, resilience, and cognitive ability. Based on this, this study used the extended broaden-and-build theory of positive emotions to explain the theoretical presuppositions among physical activity, emotional intelligence, resilience, and regulatory emotional self-efficacy.

### Practical implications

Studies have showed that regulatory emotional self-efficacy is strongly associated with prosocial behaviors, low aggressive behavior problems, and low anxiety and depression problems (Caprara et al., 2008). This is very consistent with previous studies. The study by Sui et al. (2021). has showed that regulatory emotional self-efficacy is an important mediator explaining the impact of nurses’ personalities on COVID-19 pandemic-related negative emotions. However, unlike previous studies showing that physical activity can have a direct positive impact on self-efficacy, the positive effects of physical activity on emotion-regulating self-efficacy in this study need to be mediated entirely by mental resilience and emotional intelligence. This further demonstrates the importance of positive emotions for the establishment of an individual’s emotional capacity; building an individual’s emotional competence requires a process, and the positive impact of physical activity on an individual’s dynamic ability needs to be transmitted through positive emotions, rather than directly affected by subjective feelings. This study demonstrates the positive effect of nurses’ physical activity on regulatory emotional self-efficacy and the mechanism of action in the process. Therefore, the broader promotional effect of physical activity on an individual’s physical and psychological levels deserves attention. For example, in the early days of COVID-19, in Wuhan, China, the epicenter of the outbreak, nurses and patients in field hospitals used exercise to reduce fatigue and enhance health. During the COVID-19 pandemic in Shanghai in the spring of 2022, exercise was also widely used by nurses and patients in various field hospitals. During the COVID-19 pandemic, nurses are often under tremendous physical and mental pressure as frontline workers in epidemic prevention and control. On the one hand, changes in the work environment and increased workload often make it difficult for them to adapt in a short period, which in turn creates physical and psychological stress (Alsaqri et al., 2021). On the other hand, as mentioned above, the COVID-19 pandemic has changed nurses’ work content, and the daily repetitive nucleic acid testing work has become their daily work. The dull and single-work content cannot give them get fun and a sense of achievement from their work. Meanwhile, alleviating the public’s anxiety about the COVID-19 pandemic often requires a lot of emotional labor. These factors make nurses prone to mental fatigue, which increases the risk of mental illness (Soylu et al., 2021). From the results obtained, the physical benefits obtained by physical exercise in the nurse group also positively impacted the mental health of the nurse group. On the one hand, a muscular physique helps improve the individual’s work and quality of life, and work and quality of life are the premises of ensuring the individual’s mental health. On the other hand, the positive emotional experience obtained by the individual in physical activity can effectively relieve the emotional pressure generated by the individual, and the individual can enhance the emotional ability in the long-term positive emotional experience, which represents the enhancement of the individual’s resistance to negative emotions, thereby improving the individual’s mental health.

However, despite the proven benefits of participating in physical activity, the level of physical participation of the public and special health-care workers is actually not high. This result can be attributed to the following reasons. The public does not have a clear understanding of the benefits of sports participation. The conditions for providing public participation in sports activities, including venues, time, and sports instructors, are not sufficient. The atmosphere of public participation in sports is not strong enough. Based on this, this study puts forward the following suggestions for the nurses. To improve the awareness of nurses’ participation in sports, the government and hospital management departments should carry out more publicity activities about the public’s participation in sports activities (including the benefits of sports, exercise tips, exercise precautions). Hospitals should encourage nurses to develop the habit of participating in sports every day and provide conditions such as venue and time for nurses to participate in sports activities. Sports activities should be regarded by hospitals as one of the most important components of cultural construction, and the purpose of improving nurses’ participation in sports atmosphere is achieved by regularly organizing collective sports activities.

### Limitations

There are certain limitations to this study. This study only selected two mediating variables, emotional intelligence, and resilience, to explore the impact mechanism of physical activity on regulatory emotional self-efficacy. More variables should be involved in future research. Different ages and genders have different performances on regulatory emotional self-efficacy, and future research can subdivide the effect of physical activity on regulatory emotional self-efficacy on age and gender.

## Conclusion

This study confirmed the positive effect of physical activity on the regulatory emotional self-efficacy of nurses. Specifically, the effect of physical activity on regulatory emotional self-efficacy was mediated by two variables: emotional intelligence and resilience. In the face of the severe pandemic in the world, nurses are often the type of group that suffers from greater physical and mental pressures. Considering that the current level of nurses’ participation in sports is low, the government and hospital management departments should take corresponding measures to increase the level of physical activity of nurses.

## Data availability statement

The raw data supporting the conclusions of this article will be made available by the authors, without undue reservation.

## Ethics statement

The studies involving human participants were reviewed and approved by the study was approved by the Ethics Committee of the School of Physical Education of Hunan University of Science and Technology (No. ECSPEHUST 2022/0010). The patients/participants provided their written informed consent to participate in this study.

## Author contributions

RW and HW contributed to conception and design of the study. RW and LJ organized the database. RW and JY performed the statistical analysis. RW wrote the first draft of the manuscript. LJ, YL, HW, and JY wrote sections of the manuscript. All authors contributed to the article and approved the submitted version.

## Funding

This study was supported by the Hunan Provincial Social Science Committee (No. XSP21YBZ163) and the Scientific Research Fund of Hunan University of Science and Technology (No. E52203).

## Conflict of interest

The authors declare that the research was conducted in the absence of any commercial or financial relationships that could be construed as a potential conflict of interest.

## Publisher’s note

All claims expressed in this article are solely those of the authors and do not necessarily represent those of their affiliated organizations, or those of the publisher, the editors and the reviewers. Any product that may be evaluated in this article, or claim that may be made by its manufacturer, is not guaranteed or endorsed by the publisher.

## References

[ref1] Akbari A. Khormaiee F. (2015). The prediction of mediating role of resilience between psychological well-being and emotional intelligence in students. Int. J. Public Health 2, 1–5. doi: 10.17795/intjsh-26238

[ref2] Alsaqri S. Pangket P. Alkuwaisi M. Llego J. Alshammari M. (2021). COVID-19 associated social stigma as experienced by frontline nurses of hail: a qualitative study. Int. J. Adv. Appl Sci. 8, 52–57. doi: 10.21833/ijaas.2021.08.007

[ref3] Andersen L. G. Groenvold M. Jørgensen T. Aadahl M. (2010). Construct validity of a revised physical activity scale and testing by cognitive interviewing. Scand. J. Public Health 38, 707–714. doi: 10.1177/1403494810380099, PMID: 20823047

[ref4] Anderson J. C. Gerbing D. W. (1988). Structural equation modeling in practice: a review and recommended two-step approach. Psychol. Bull. 103, 411–423. doi: 10.1037/0033-2909.103.3.411

[ref5] Aouani H. Slimani M. Ghouili H. Tod D. Znazen H. Bragazzi N. L.. (2022). Emotional intelligence: a systematic comparison between Young athletes and non-athletes, gender and age groups. Int. J. Sport. Stud. Hlth. 5:e128656. doi: 10.5812/intjssh-128656

[ref6] Bandura A. Caprara G. V. Barbaranelli C. Gerbino M. Pastorelli C. (2003). Role of affective self-regulatory efficacy in diverse spheres of psychosocial functioning. Child Dev. 74, 769–782. doi: 10.1111/1467-8624.00567, PMID: 12795389

[ref7] Bollen K. A. Stine R. (1990). Direct and indirect effects: classical and bootstrap estimates of variability. Sociol. Methodol. 20, 115–140. doi: 10.2307/271084

[ref8] Bouchard C. Shephard R. J. Brubaker P. H. (1994). Physical activity, fitness, and health: consensus statement. Med. Sci. Sports Exerc. 26:119. doi: 10.1249/00005768-199401000-00024

[ref9] Campbell-Sills L. Stein M. B. (2007). Psychometric analysis and refinement of the Connor-davidson resilience scale (CD-RISC): validation of a 10-item measure of resilience. J. Trauma. Stress. 20, 1019–1028. doi: 10.1002/jts.20271, PMID: 18157881

[ref10] Caprara G. V. Di Giunta L. Eisenberg N. Gerbino M. Pastorelli C. Tramontano C. (2008). Assessing regulatory emotional self-efficacy in three countries. Psychol. Assess. 20, 227–237. doi: 10.1037/1040-3590.20.3.227, PMID: 18778159 PMC2713723

[ref11] Caprara G. V. Steca P. Cervone D. Artistico D. (2003). The contribution of self-efficacy beliefs to dispositional shyness: on social-cognitive systems and the development of personality dispositions. J. Pers. Soc. Psychol. 71, 943–970. doi: 10.1111/1467-6494.7106003, PMID: 14633054

[ref12] Chan D. W. (2004). Perceived emotional intelligence and self-efficacy among Chinese secondary school teachers in Hong Kong. Pers. Individ. Dif. 36, 1781–1795. doi: 10.1016/j.paid.2003.07.007

[ref13] Chan A. O. Chan Y. H. Kee J. P. (2013). Exposure to crises and resiliency of health care workers in Singapore. Occup. Med. (Chic. Ill.). 63, 141–144. doi: 10.1093/occmed/kqs202, PMID: 23223749

[ref14] Chekroud S. R. Gueorguieva R. Zheutlin A. B. Paulus M. Krumholz H. M. Krystal J. H.. (2018). Association between physical exercise and mental health in 1·2 million individuals in the USA between 2011 and 2015: a cross-sectional study. Lancet Psychiatry 5, 739–746. doi: 10.1016/S2215-0366(18)30227-X, PMID: 30099000

[ref15] Ciarrochi J. Deane F. P. Anderson S. (2002). Emotional intelligence moderates the relationship between stress and mental health. Pers. Individ. Dif. 32, 197–209. doi: 10.1016/S0191-8869(01)00012-5

[ref17] Fino E. Bonfrate I. Fino V. Bocus P. Russo P. M. Mazzetti M. (2021). Harnessing distress to boost growth in frontline healthcare workers during COVID-19 pandemic: the protective role of resilience, emotion regulation and social support. Appl. Nurs. Res., 1–3. doi: 10.1017/S0033291721000519, PMID: 33565390 PMC7900668

[ref18] Fornell C. Larcker D. F. (1981). Evaluating structural equation models with unobservable variables and measurement error. J. Mark. Res. 18, 39–50. doi: 10.1177/002224378101800104

[ref19] Frazier S. E. Nagy S. (1989). Mood state changes of women as a function of regular aerobic exercise. Percept. Mot. Skills 68, 283–287. doi: 10.2466/pms.1989.68.1.283, PMID: 2928059

[ref20] Fredrickson B. L. (1998). What good are positive emotions? Rev. Gen. Psychol. 2, 300–319. doi: 10.1037/1089-2680.2.3.300, PMID: 21850154 PMC3156001

[ref21] Fredrickson B. L. (2001). The role of positive emotions in positive psychology: the broaden-and-build theory of positive emotions. Am. Psychol. 56, 218–226. doi: 10.1037/0003-066X.56.3.218 11315248 PMC3122271

[ref22] Gehlhar A. Schmidt N. Eisenburger N. Feddern S. Kossow A. Nießen J.. (2022). Impact of physical activity on COVID-19-related symptoms and perception of physical performance, fatigue and exhaustion during stay-at-home orders. BMJ Open Sport Exerc. Med. 8:e001319. doi: 10.1136/bmjsem-2022-001319, PMID: 35539285 PMC9072782

[ref23] Hair J. F. Sarstedt M. Ringle C. M. Mena J. A. (2012). An assessment of the use of partial least squares structural equation modeling in marketing research. J. Acad. Mark. Sci. 40, 414–433. doi: 10.1007/s11747-011-0261-6

[ref24] Hamre K. V. Einarsen S. V. Hoprekstad Ø. L. Pallesen S. Bjorvatn B. Waage S.. (2020). Accumulated long-term exposure to workplace bullying impairs psychological hardiness: a five-year longitudinal study among nurses. Int. J. Environ. Res. Public Health 17:2587. doi: 10.3390/ijerph17072587, PMID: 32290042 PMC7178264

[ref25] Jackson D. L. Gillaspy J. A. Purc-Stephenson R. (2009). Reporting practices in confirmatory factor analysis: an overview and some recommendations. Psychol. Methods 14, 6–23. doi: 10.1037/a0014694, PMID: 19271845

[ref26] Jayalakshmi V. Magdalin S. (2015). Emotional intelligence, resilience and mental health of women college students. J. Psychol. Res. 10, 401–408.

[ref27] Jeung D.-Y. Kim C. Chang S.-J. (2018). Emotional labor and burnout: a review of the literature. Yonsei Med. J. 59, 187–193. doi: 10.3349/ymj.2018.59.2.187, PMID: 29436185 PMC5823819

[ref28] Kavanagh D. J. Bower G. H. (1985). Mood and self-efficacy: impact of joy and sadness on perceived capabilities. Cognit. Ther. Res. 9, 507–525. doi: 10.1007/BF01173005

[ref29] Keshkar S. Dickson G. Ahonen A. Swart K. Addesa F. Epstein A.. (2021). The effects of coronavirus pandemic on the sports industry: an update. Ann. Appl. Sport. Sci. 9, 01–10. doi: 10.29252/aassjournal.964

[ref30] Kimberlin C. L. Winterstein A. G. (2008). Validity and reliability of measurement instruments used in research. Am. J. Health Syst. Pharm. 65, 2276–2284. doi: 10.2146/ajhp070364 19020196

[ref31] Kumpfer K. L. (2002). “Factors and processes contributing to resilience” in Resilience and Development: Positive Life Adaptations. eds. Glantz M. D. Johnson J. L. (Boston, MA: Springer), 179–224.

[ref32] Labrague L. J. De los Santos J. A. A. (2020). COVID-19 anxiety among front-line nurses: predictive role of organisational support, personal resilience and social support. J. Nurs. Manag. 28, 1653–1661. doi: 10.1111/jonm.13121, PMID: 32770780 PMC7436313

[ref33] Law K. S. Wong C.-S. Song L. J. (2004). The construct and criterion validity of emotional intelligence and its potential utility for management studies. J. Appl. Psychol. 89, 483–496. doi: 10.1037/0021-9010.89.3.483, PMID: 15161407

[ref34] Liu J. Qiang F. (2022). Psychosocial mediation of light-moderate physical activity cognitive performance among adults aged 60+ in China, China. Behav. Sci. 12:967. doi: 10.3390/bs12060175 PMC9220284 35735384

[ref35] Lorente L. Vera M. Peiró T. (2021). Nurses stressors and psychological distress during the COVID-19 pandemic: the mediating role of coping and resilience. J. Adv. Nurs. 77, 1335–1344. 33210768 10.1111/jan.14695 PMC7753515

[ref36] Masten A. S. (2001). Ordinary magic: resilience processes in development. Am. Psychol. 56, 227–238. doi: 10.1037/0003-066X.56.3.227, PMID: 11315249

[ref37] Masten A. S. Coatsworth J. D. (1998). The development of competence in favorable and unfavorable environments: lessons from research on successful children. Am. Psychol. 53, 205–220. doi: 10.1037/0003-066X.53.2.205, PMID: 9491748

[ref38] Mayer J. D. (2015). The personality systems framework: current theory and development. J. Res. Pers. 56, 4–14. doi: 10.1016/j.jrp.2015.01.001

[ref39] Mayer J. D. Caruso D. R. Salovey P. (1999). Emotional intelligence meets traditional standards for an intelligence. Intelligence 27, 267–298. doi: 10.1016/S0160-2896(99)00016-1

[ref40] McEwen B. S. Gray J. D. Nasca C. (2015). 60 Years of neuroendocrinology: redefining neuroendocrinology: stress, sex and cognitive and emotional regulation. J. Endocrinol. 226, T67–T83. doi: 10.1530/JOE-15-0121, PMID: 25934706 PMC4515381

[ref16] National Health Commission (2022). *2020 Statistical Bulletin of China’s Health Development*. Available online: http://www.gov.cn/guoqing/2021-07/22/content_5626526.htm (Accessed June 14, 2022).

[ref41] Paluska S. A. Schwenk T. L. (2000). Physical activity and mental health. Sports Med. 29, 167–180. doi: 10.2165/00007256-200029030-00003 10739267

[ref42] Salovey P. Mayer J. Caruso D. Yoo S. H. (2009). “The positive psychology of emotional intelligence” in Oxford Handbook of Positive Psychology. eds. Snyder C. R. Lopez S. J. (Oxford: Oxford University Press), 237–248.

[ref43] Scully D. Kremer J. Meade M. M. Graham R. Dudgeon K. (1998). Physical exercise and psychological well being: a critical review. Br. J. Sports Med. 32, 111–120. doi: 10.1136/bjsm.32.2.111, PMID: 9631216 PMC1756084

[ref44] Sharma J. Dhar R. L. Tyagi A. (2016). Stress as a mediator between work–family conflict and psychological health among the nursing staff: moderating role of emotional intelligence. Appl. Nurs. Res. 30, 268–275. doi: 10.1016/j.apnr.2015.01.010, PMID: 25769936

[ref45] Smith P. A. Gould M. M. See Tai S. Iliffe S. (1996). Exercise as therapy? Results from group interviews with general practice teams involved in an inner-London 'prescription for exercise' scheme. Health Educ. J. 55, 439–446. doi: 10.1177/001789699605500409

[ref46] Soylu Y. Arslan E. Kilit B. (2021). Psychophysiological responses and cognitive performance: a systematic review of mental fatigue on soccer performance. Int. J. Sport. Stud. Hlth. 4:e124244. doi: 10.5812/intjssh.124244

[ref47] Stafseth S. K. Skogstad L. Ræder J. Hovland I. S. Hovde H. Ekeberg Ø.. (2022). Symptoms of anxiety, depression, and post-traumatic stress disorder in health care personnel in Norwegian ICUs during the first wave of the COVID-19 pandemic, a prospective, observational cross-sectional study. Int. J. Environ. Res. Public Health 19:7010. doi: 10.3390/ijerph19127010, PMID: 35742259 PMC9222786

[ref48] Sui W. Gong X. Zhuang Y. (2021). The mediating role of regulatory emotional self-efficacy on negative emotions during the COVID-19 pandemic: a cross-sectional study. Int. J. Ment. Health Nurs. 30, 759–771. doi: 10.1111/inm.12830, PMID: 33586868 PMC8013741

[ref49] Tamminen K. A. Bennett E. V. (2017). No emotion is an island: an overview of theoretical perspectives and narrative research on emotions in sport and physical activity. Qual. Res. In sport. Exerc and Health. 9, 183–199. doi: 10.1080/2159676X.2016.1254109

[ref50] Tugade M. M. Fredrickson B. L. (2004). Resilient individuals use positive emotions to bounce back from negative emotional experiences. J. Pers. Soc. Psychol. 86, 320–333. doi: 10.1037/0022-3514.86.2.320, PMID: 14769087 PMC3132556

[ref51] Wang K. Yang Y. Zhang T. Ouyang Y. Liu B. Luo J. (2020). The relationship between physical activity and emotional intelligence in college students: the mediating role of self-efficacy. Front. Psychol. 11:967. doi: 10.3389/fpsyg.2020.00967 32581908 PMC7296084

[ref52] Young J. L. Derr D. M. Cicchillo V. J. Bressler S. (2011). Compassion satisfaction, burnout, and secondary traumatic stress in heart and vascular nurses. Crit. Care Nurs. Q. 34, 227–234. doi: 10.1097/CNQ.0b013e31821c67d5, PMID: 21670622

